# Receptor density pattern confirms and enhances the anatomic-functional features of the macaque superior parietal lobule areas

**DOI:** 10.1007/s00429-019-01930-9

**Published:** 2019-08-07

**Authors:** Daniele Impieri, Karl Zilles, Meiqi Niu, Lucija Rapan, Nicole Schubert, Claudio Galletti, Nicola Palomero-Gallagher

**Affiliations:** 1 Department of Biomedical and Neuromotor Sciences, University of Bologna, 40126 Bologna, Italy; 2 Institute of Neuroscience and Medicine (INM-1), Research Centre Jülich, 52425 Jülich, Germany; 3 JARA-BRAIN, Jülich-Aachen Research Alliance, Jülich, Germany; 4 Department of Psychiatry, Psychotherapy and Psychosomatics, Medical Faculty, RWTH, Aachen, Germany

**Keywords:** Monkey, Neurochemical organization, Posterior parietal cortex, Somatosensory-motor input, Visuo-motor input

## Abstract

The macaque monkey superior parietal lobule (SPL) is part of a neuronal network involved in the integration of information from visual and somatosensory cortical areas for execution of reaching and grasping movements. We applied quantitative in vitro receptor autoradiography to analyse the distribution patterns of 15 different receptors for glutamate, GABA, acetylcholine, serotonin, dopamine, and adenosine in the SPL of three adult male *Macaca fascicularis* monkeys. For each area, mean (averaged over all cortical layers) receptor densities were visualized as a receptor fingerprint of that area. Multivariate analyses were conducted to detect clusters of areas according to the degree of (dis)similarity of their receptor organization. Differences in regional and laminar receptor distributions confirm the location and extent of areas V6, V6Av, V6Ad, PEc, PEci, and PGm as found in cytoarchitectonic and functional studies, but also enable the definition of three subdivisions within area PE. Receptor densities are higher in supra- than in infragranular layers, with the exception of kainate, M_2_, and adenosine receptors. Glutamate and GABAergic receptors are the most expressed in all areas analysed. Hierarchical cluster analyses demonstrate that SPL areas are organized in two groups, an organization that corresponds to the visual or sensory-motor characteristics of those areas. Finally, based on present results and in the framework of our current understanding of the structural and functional organization of the primate SPL, we propose a novel pattern of homologies between human and macaque SPL areas.

## Introduction

The movement of the limbs necessary to reach or grasp objects requires the integration in the cerebral cortex of motor signals with visual and somatosensory stimuli. The brain regions activated during the association of these different sensory-motor modalities to enable the execution of more or less fine movements of the limbs are known as associative cortices.

**Fig. 1:**
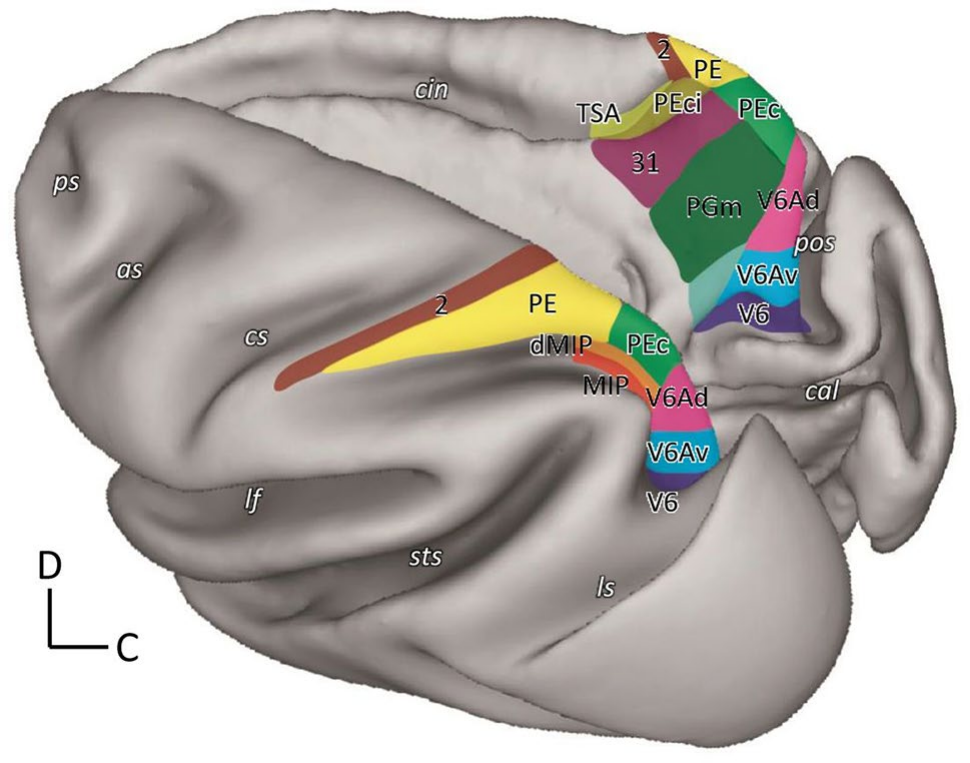
Location and extent of SPL and adjoining areas in the macaque brain. 3D reconstruction of the left hemisphere (in dorsal view) and of the right one (in mesial view) of a macaque monkey brain obtained using CARET software (http://brainvis.wustl.edu/wiki/index.php/Caret:Download) showing the location and extent of the areas that compose the SPL, as well as of directly adjacent areas. *as* arcuate sulcus, *cal* calcarine sulcus, *cin* cingulate sulcus, *cs* central sulcus, *ips* intraparietal sulcus, *lf* lateral fissure, *ls* lunate sulcus, *ps* principal sulcus, *pos* parieto-occipital sulcus, *sts* superior temporal sulcus, *C* caudal, *D* dorsal

The superior parietal lobule (SPL) of the macaque monkey is recognized as an associative cortex, as it is part of a neuronal network involved in the association of information coming from frontal and visual cortices useful to plan and control the execution of reaching and grasping movements (Galletti and Fattori 2018; Galletti et al. 2003; Goodale and Milner 1992; Rizzolatti and Matelli 2003). This brain region hosts several cyto- and myelo-architectonically defined areas (Colby et al. 1988; Luppino et al. 2005; Pandya and Seltzer 1982; see Fig. 1), some of which to date have also been electrophysiologically extensively investigated, but others much less studied.

Functional and anatomical studies of SPL areas showed that there are two opposite but heavily interconnected flows of information within this structure, a visual one directed caudo-rostrally and a somatosensory one rostro-caudally, with a strong sensory-motor integration in between. Indeed, moving anteriorly from the posterior end of SPL, we find areas V6 (pure visual motion area; Galletti et al. 1999a, 2001; Gamberini et al. 2015), V6Av and V6Ad (visuo-motor areas with somatosensory influence from the upper limbs; Galletti et al. 1999b; Gamberini et al. 2009, 2011, 2015, 2018; Passarelli et al. 2011), PEc (visuo-motor area with higher incidence of the somatosensory input from both upper and lower limbs; Bakola et al. 2010; Breveglieri et al., 2006, 2008; Gamberini et al. 2018; Piserchia et al. 2017), and PE (somatosensory-motor area without visual influence; Bakola et al. 2013; Mountcastle et al. 1975; Padberg et al. 2007; Seelke et al. 2012). Other areas seem to have a corollary role in this sensory-motor network, as area PGm is mainly involved in oculomotor activity, spatial navigation (Leichnetz 2001; Olson et al. 1996; Passarelli et al. 2018; Thier and Andersen 1998), and visually guided limb movements (Ferraina et al. 1997; Passarelli et al. 2018), and area PEci displays sensory-motor properties (Morecraft et al. 2004; Murray and Coulter 1981).

The aim of this work is to define the chemo-architecture of the cortex of the SPL, looking for possible correlations between cytoarchitectonic patterns, chemoarchitectonic organization, and functional segregations of the areas located therein.

## Materials and methods

All the experimental protocols were in accordance with the guidelines of the European laws for the care and use of animals for scientific purposes.

Four hemispheres of three *Macaca fascicularis* brains were used to collect the data shown in this study (animal ID #11539, left and right hemispheres; animal ID #11543, left hemisphere; animal ID #11530, left hemisphere). All the animals were male specimens between 6 and 8 years old, with a body weight between 5.2 and 6.6 kg, and obtained from Covance Company, Münster, where they were used as control animals for pharmaceutical studies performed in compliance with legal requirements.

### Histological procedures

The animals were killed receiving a lethal dose of sodium pentobarbital (i.v. injection). Then, the brains were removed from the skull, and brain stem and cerebellum were dissected off in close proximity to the cerebral peduncles. The brains were divided into hemispheres cutting the corpus callosum, and then in a rostral and a caudal block making a cut in coronal plane of sectioning between the central and arcuate sulci. The unfixed tissue blocks were frozen in isopentane at − 40 to − 50 °C, and then stored in airtight plastic bags at − 70 °C. Each block was then sectioned in the coronal plane using a cryostat microtome (CM 3050, Leica, Germany), obtaining slices of 20 µm thickness which were thaw-mounted on gelatine-coated slides and freeze-dried overnight. Alternating sections were stained for cell bodies (Merker 1983) or myelin (Gallyas 1979), or processed for the visualization of neurotransmitter receptor binding sites. Specifically, tissue blocks were serially sectioned in such a way that groups of 25 sections (“repeats”) were collected throughout the slab, and 20 sections were discarded between repeats. Repeats consisted of a predetermined order of sections meant for the visualization of a specific receptor type or histological staining. Every 4th and 15th sections of a repeat were used for visualization of cell bodies, and every 9th and 20th sections for the myelin stain. Thus, the distance between two sections processed for the same receptor type was of 900 µm. At six different rostrocaudal levels throughout the brain, the “repeat” consisted of 46 sections, since it also included sections used for the determination of non-specific binding (see below).

### Receptor autoradiographic labelling

Quantitative in vitro receptor autoradiography was applied to label 15 different receptors for the transmitters glutamate (AMPA, kainate, NMDA), GABA (GABA_A_, GABA_B_, GABA_A_ associated benzodiazepine [GABA_A_/BZ] binding sites), acetylcholine (muscarinic M_1_, M_2_, M_3_), noradrenaline (α_1_, α_2_), serotonin (5-HT_1A_, 5-HT_2_), dopamine (D_1_), and adenosine (A_1_) by incubating the sections in solutions of respective tritiated ligands according to previously published protocols (Palomero-Gallagher and Zilles 2018; Zilles et al. 2002). Incubation protocols are specified in Table 1. In short, the labelling protocol included a washing step to rehydrate the sections and remove endogenous substances, a main incubation, and a final rinsing step to remove the surplus ligand. In the main incubation, sections were incubated with either a tritiated ligand alone (in nm concentrations) to determine total binding, or with the tritiated ligand (also in nm concentrations) accompanied by a non-labelled specific displacer (in µm concentrations) to determine the proportion of displaceable, non-specific binding. Specific binding is the difference between total and non-specific binding. Since the ligands and binding protocols used resulted in a displaceable binding, which was less than 5% of the total binding, we consider total binding to be equivalent of specific binding. The sections processed for receptor autoradiography were then exposed together with plastic samples of known radioactivity against tritium-sensitive films (Hyperfilm, Amersham) for a period of 4–12 weeks based on the ligand used.

**Table 1:** Incubation protocols

Transmitter	Receptor	Ligand (nM)	Displacer	Incubation buffer	Pre-incubation	Main incubation	Final rinsing
Glutamate	AMPA	[^3^H]-AMPA (10.0)	Quisqualate (10 μM)	50 mM Tris–acetate (pH 7.2) [+ 100 mM KSCN]*	3 × 10 min, 4 °C	45 min, 4 °C	4 × 4 s Acetone/glutaraldehyde (100 ml + 2.5 ml), 2 × 2 s, 4 °C
	NMDA	[^3^H]-MK-801 (3.3)	(+)MK-801 (100 μM)	50 mM Tris–acetate (pH 7.2) + 50 μM glutmate [+ 30 μM glycine + 50 μM spermidine]*	15 min, 4 °C	60 min, 22 °C	2 × 5 min, 4 °C Distilled water, 1 × 22 °C
	Kainate	[^3^H]-Kainate (9.4)	SYM 2081 (100 μM)	50 mM Tris–acetate (pH 7.2) [+ 10 mM Ca^2+^-acetate]*	3 × 10 min, 4 °C	45 min, 4 °C	3 × 4 s Acetone/glutaraldehyde (100 ml + 2.5 ml), 2 × 2 s, 22 °C
GABA	GABA_A_	[^3^H]-Muscimol (7.7)	GABA (10 μM)	50 mM Tris–citrate (pH 7.0)	3 × 5 min, 4 °C	40 min, 4 °C	3 × 3 s, 4 °C Distilled water, 1 × 22 °C
	GABA_B_	[^3^H]-CGP 54626 (2.0)	CGP 55845 (100 μM)	50 mM Tris–HCl (pH 7.2) + 2.5 mM CaCl_2_	3 × 5 min, 4 °C	60 min, 4 °C	3 × 2 s, 4 °C Distilled water, 1 × 22 °C
	Benzodiazepine binding site	[^3^H]-Flumazenil (1.0)	Clonazepam (2 μM)	170 mM Tris–HCl (pH 7.4)	15 min, 4 °C	60 min, 4 °C	2 × 1 min, 4 °C Distilled water, 1 × 22 °C
Acetylcholine	M_1_	[^3^H]-Pirenzepine (1.0)	Pirenzepine (2 μM)	Modified Krebs buffer (pH 7.4)	15 min, 4 °C	60 min, 4 °C	2 × 1 min, 4 °C Distilled water, 1 × 22 °C
	M_2_	[^3^H]-Oxotremorine-M (1.7)	Carbacol (10 μM)	20 mM HEPES-Tris (pH 7.5) + 10 mM MgCl_2_ + 300 nM Pirenzepine	20 min, 22 °C	60 min, 22 °C	2 × 2 min, 4 °C Distilled water, 1 × 22 °C
	M_3_	[^3^H]-4-DAMP (1.0)	Atropine sulfate (10 μM)	50 mM Tris–HCl (pH 7.4) + 0.1 mM PSMF + 1 mM EDTA	15 min, 22° C	45 min, 22° C	2 × 5 min, 4°C Distilled water, 1 × 22°C
Noradrenaline	α_1_	[^3^H]-Prazosin (0.2)	Phentolamine mesylate (10 μM)	50 mM Na/K-phosphate buffer (pH 7.4)	15 min, 22 °C	60 min, 22 °C	2 × 5 min, 4 °C Distilled water, 1 × 22 °C
	α_2_	[^3^H]-UK 14,304 (0,64)	Phentolamine mesylate (10 μM)	50 mM Tris–HCl + 100 μM MnCl_2_ (pH 7.7)	15 min, 22 °C	90 min, 22 °C	5 min, 4 °C Distilled water, 1 × 22 °C
Serotonin	5-HT_1A_	[^3^H]-8-OH-DPAT (1.0)	5-Hydroxy-tryptamine (1 μM)	170 mM Tris–HCl (pH 7.4) [+ 4 mM CaCl_2_ + 0.01% ascorbate]*	30 min, 22 °C	60 min, 22 °C	5 min, 4 °C Distilled water, 3 × 22 °C
	5-HT_2_	[^3^H]-Ketanserin (0.5)	Mianserin (10 μM)	170 mM Tris–HCl (pH 7.7)	30 min, 22 °C	120 min, 22 °C	2 × 10 min, 4 °C Distilled water, 3 × 22 °C
Dopamine	D_1_	[^3^H]-SCH 23390 (1.67)	SKF 83566 (1 μM)	50 mM Tris–HCl + 120 mM NaCl + 5 mM KCl + 2 mM CaCl_2_ + 1 mM MgCl_2_ (pH 7.4)	20 min, 22 °C	90 min, 22 °C	2 × 20 min, 4 °C Distilled water, 1 × 22 °C
Adenosine	A_1_	[^3^H]-DPCPX (1.0)	R-PIA (100 μM)	170 mM Tris–HCl + 2 Units/I Adenosine deaminase [+ 100 μM Gpp(NH)p]* (pH 7.4)	15 min, 4 °C	120 min, 22 °C	2 × 5 min, 4 °C Distilled water, 1 × 22 °C

*Only included in the main incubation

### Image analysis

The ensuing autoradiographs were processed by densitometry with a video-based image analysing technique described in previously published studies (Palomero-Gallagher and Zilles 2018; Zilles et al. 2002). Briefly, the autoradiographs were digitized using a CCD camera, and stored as 8-bit grey value images. The plastic scales of known radioactivity were used to create a transformation curve to linearize the autoradiographs, i.e. to transform the grey values in each pixel of the autoradiograph into concentrations of radioactivity in the tissue. These concentrations of radioactivity were then converted into binding-site densities, *B*_max_ values (concentration values in fmol/mg protein at saturation of the ligand–receptor complex) by multiplying the grey values of the linearized autoradiographs by (*K*_D_ + *c*)/*c* (where *K*_D_ is a dissociation constant of the ligand–receptor binding kinetics at the equilibrium phase, and *c* the free concentration of labelled ligand in the incubation buffer). Additionally, linearized autoradiographs were subjected to linear contrast enhancement, colour coding and median filtering for visualization purposes. These final steps were useful to obtain images that could be analysed by visual inspection, to subdivide the SPL into different cortical areas.

The mean areal density value for each area was calculated using in house software (AnaRec), which extracted the mean of the grey values contained in a specific cortical area and transformed it into a receptor concentration per unit protein (fmol/mg protein). To this purpose, and depending on the size of the area, a series of 3–5 equidistantly spaced sections per animal and receptor type were analysed. The ensuing receptor densities were represented as multi-receptor fingerprints, i.e. as polar coordinate plots simultaneously depicting the concentrations of all examined receptor types within a given cortical area. After that, all the data available were analysed to obtain a “receptor fingerprint” for each identified cortical area.

For each identified area, a Grey Level Index (GLI) value was also obtained from sections stained with the Nissl method to quantitatively compare the cytoarchitecture of the areas examined in this study (Wree et al. 1982). This analysis was performed choosing the best cytological segment of each cortical area, where the plane of sectioning was perpendicular to all cortical layers. The GLI, which quantifies the volume of cell bodies relative to the total brain volume, was computed using in-house MATLAB scripts (for further details, see Palomero-Gallagher and Zilles 2018; Zilles et al. 2002).

### Statistical analysis

Hierarchical cluster and Multi-Dimensional Scaling (MDS) analyses were carried out with Matlab (The MathWorks, Inc., Natick, MA) as previously described (Palomero-Gallagher et al. 2009) to determine the degree of (dis)similarity of the receptor fingerprints of SPL areas. The number of stabile clusters was determined by a subsequent *k*-means analysis and the elbow method (Rousseeuw 1987). Due to the large differences in the absolute expression levels of the different receptor types examined, receptor densities were normalized by *z*-scores prior to these analyses. In-house MATLAB scripts were also used to compute Mahalanobis distances (Mahalanobis et al. 1949) to determine the (dis)similarity in GLI values between areas of the SPL. Receptors were evaluated for possible differences between SPL areas by means of a MANOVA (*p* < 0.01). Since this was significant, we then carried out, for each receptor type separately, an ANOVA with repeated measures (*p* < 0.01; Bonferroni corrected for the 15 receptor types tested) and subsequent paired *t* tests (*p* < 0.01) to determine which area contributed to the significance. The *p* values resulting from these post-hoc tests were not corrected for multiple testing because they were only performed when the ANOVA tests had been found to be significant.

## Results

**Fig. 2:**
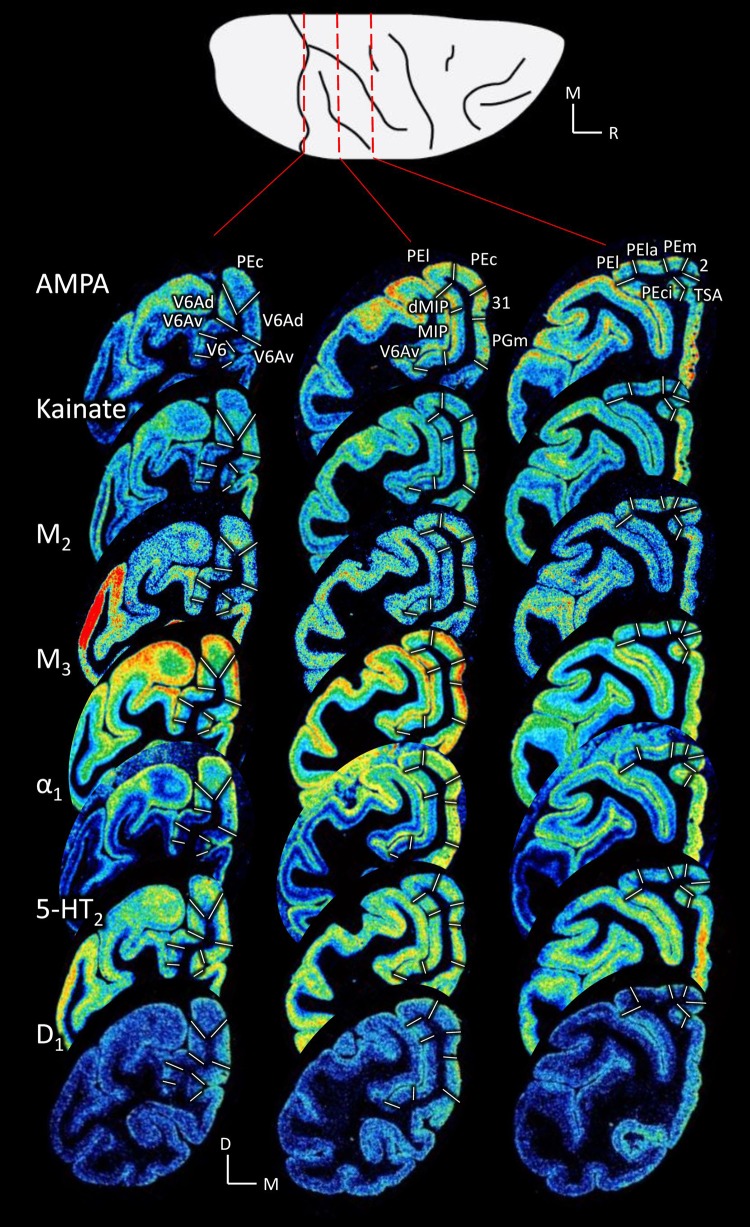
Coronal sections through three levels of a macaque hemisphere showing exemplary receptor distribution patterns in the SPL. Note the contrast between the relatively homogeneous expression of D_1_ receptors in the cerebral cortex and the heterogeneous distribution patterns of the AMPA, kainate, M_2_, M_3_, α_1_, 5-HT_2_ receptors. White lines on each section represent the borders of SPL areas. Top: silhouette of a macaque brain showing the levels from which the sections presented below were obtained. *D* dorsal, *M* mesial, *R* rostral

Fifteen different receptor types were analysed to provide insights into the molecular organization of SPL areas. These receptors are heterogeneously distributed, both at the regional and at the laminar level, throughout the cortex of the SPL. Some receptors (e.g. AMPA, kainate, M_2_, M_3_, α_1_ and 5-HT_2_; Fig. 2) were particularly useful to map the SPL, because the inter-areal differences in their expression levels clearly revealed cortical borders, whereas for other receptors (e.g. D_1_ receptor; Fig. 2), inter-areal differences were more subtle. The multimodal approach of the present study not only confirmed the existence of previously described cytoarchitectonic areas, but also enabled the definition of three subdivisions within area PE: areas PEla (or lateral-anterior PE) and PEl (lateral PE), and PEm (or medial PE).

### Cytoarchitecture of SPL areas

**Fig. 3:**
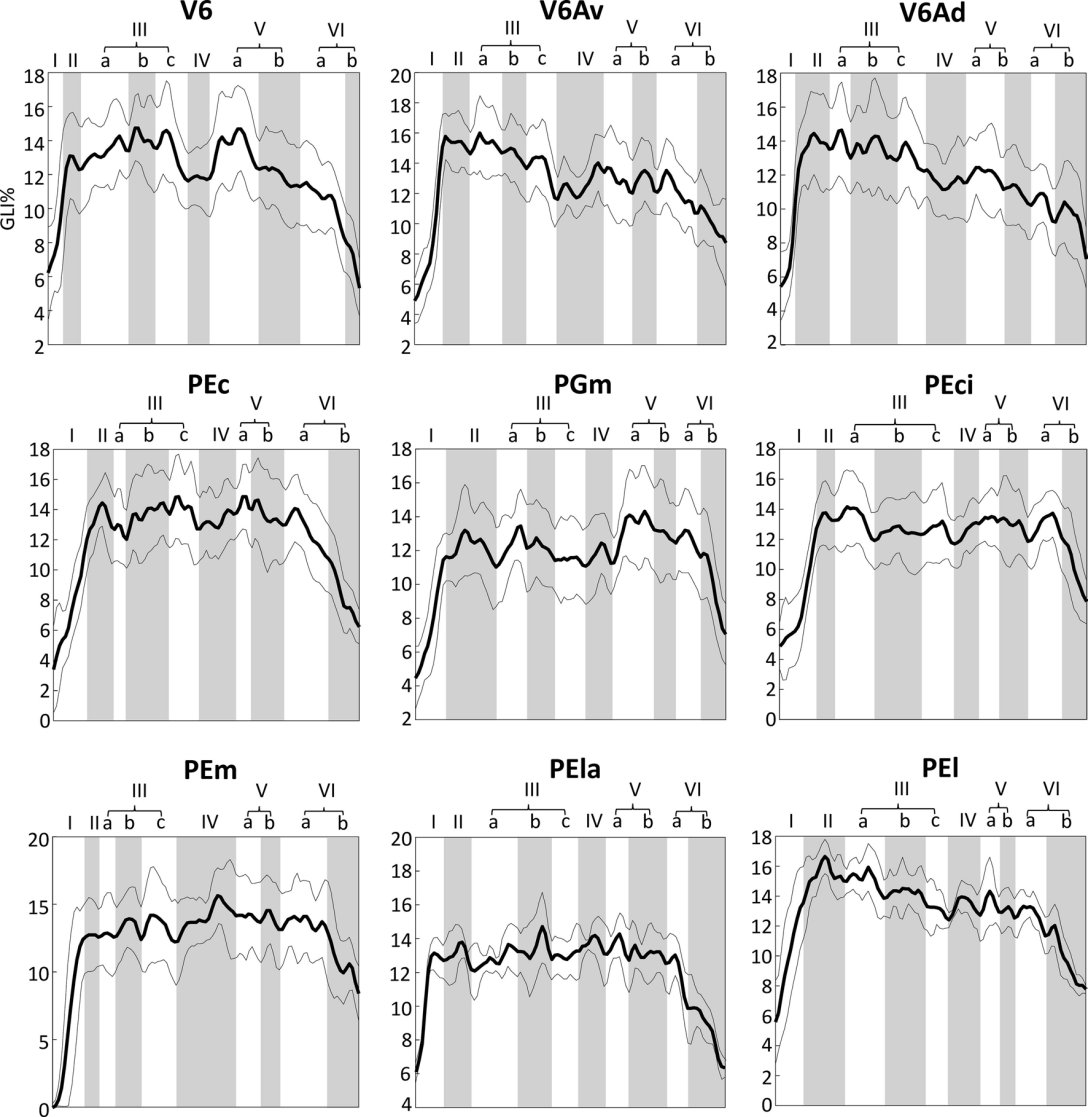
Grey Level Index (GLI) profiles quantifying the cytoarchitecture of SPL areas. They depict the mean (thick line) ± s.d. (thin lines) changes in the volume fraction of cell bodies (*y* axis) when moving prom the pial surface to the layer VI/white matter border (*x* axis)

Figure 3 shows the results obtained from the quantitative cytoarchitectonic analysis of SPL areas. The profiles represent the variations in the volume fraction of cell bodies as GLI (%) when moving from the pial surface to the border between layer VIb and the white matter. The congruity between the curve representing the mean GLI and those indicating the s.d. values highlights the ideal plane of sectioning of the site selected for GLI measurement. For all areas, a subdivision of layers III (a, b, c), V (a, b), and VI (a, b) was detected. As expected, the GLI value is low at the level of layer I, is highest between layers II and V, and then becomes low again in layer VI, particularly in layer VIb. The proportion of the thickness of each layer and sublayer changes between all the areas. As an example, area V6 shows a thinner layer IV and thicker layers IIIa and Vb in respect to the adjoining area V6Av.

**Fig. 4:**
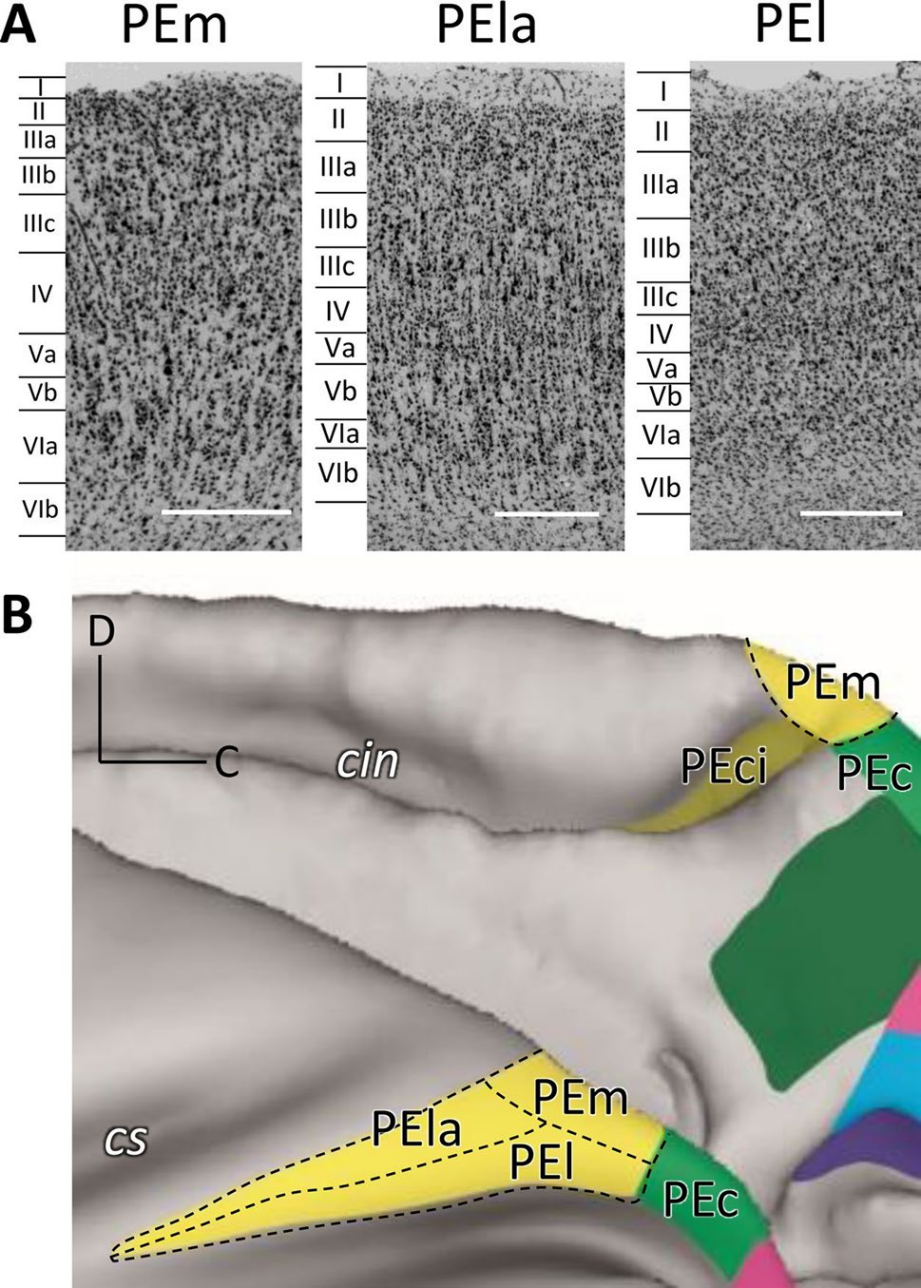
**a** Cytoarchitectonic pattern of areas PEm, PEla, and PEl. High magnification views of Nissl-stained segments of the three parts of area PE are shown. Scale bars = 500 µm. **b** Schematic visualization of the spatial relationship between the subdivisions of area PE. Other details and abbreviations as in Fig. 1

It is worthwhile to note that differences in the cytoarchitecture (Fig. 4a), as well as in the mean (densities averaged over all layers) and laminar distribution patterns of some of the examined receptors (see below), enabled the subdivision of cytoarchitectonic area PE into three portions: PEm, the dorsal most subdivision, which encroaches onto the mesial surface of the hemisphere, is abutted caudo-laterally by area PEl and rostro-laterally by area PEla (Fig. 4b). Areas PEm and PEla have a clearly visible columnar organization that is absent in area PEl. About layer thickness, the three areas have a similarly well-developed layer III; on the contrary, layer IV differs between areas: area PEm shows a thick granular layer, which becomes thinner in areas PEla and PEl. Another difference concerns the border with white matter, which is clearly distinct only in area PEla. About the cell population, area PEm shows well-stained cell bodies, with few numbers of large pyramidal cells in layer V. Area PEla, on the contrary, shows a clear strip of large pyramids in layer III, in particular in sublayer IIIc. Area PEl shows a strip of well-impregnated pyramidal neurons in correspondence of layers III. Furthermore, tissue ‘grain’ is larger, broader, and rougher in PEm, and smaller, finer and more compact in PEl, with PEla showing an intermediate pattern.

The qualitative observations from each of the examined SPL areas were confirmed by computing the Mahalanobis distances between the layer-specific GLI values extracted from each of the examined areas, as shown in Table 2. The highest degree of cytoarchitectural dissimilarities was found for area V6 with respect to PEm, and for area PEci with respect to the nearby area PEc.

**Table 2:** Matrix of Mahalanobis distances between areas of the SPL

	PEc	PEl	PEla	PEm	PEci	PGm	V6Ad	V6Av	V6
PEc	0								
PEl	5.21	0							
PEla	4.24	4.48	0						
PEm	7.39	5.15	7.97	0					
PEci	9.57	6.43	8.92	7.04	0				
PGm	7.06	6.55	5.41	7.78	4.77	0			
V6Ad	5.00	2.55	5.17	6.85	8.38	8.08	0		
V6Av	7.92	3.23	5.49	8.46	5.64	6.23	2.69	0	
V6	4.63	4.19	5.96	9.30	8.28	5.80	4.41	6.75	0

Analysis was based on the mean GLI% of each area

### Receptor-architecture of SPL areas

**Fig. 5:**
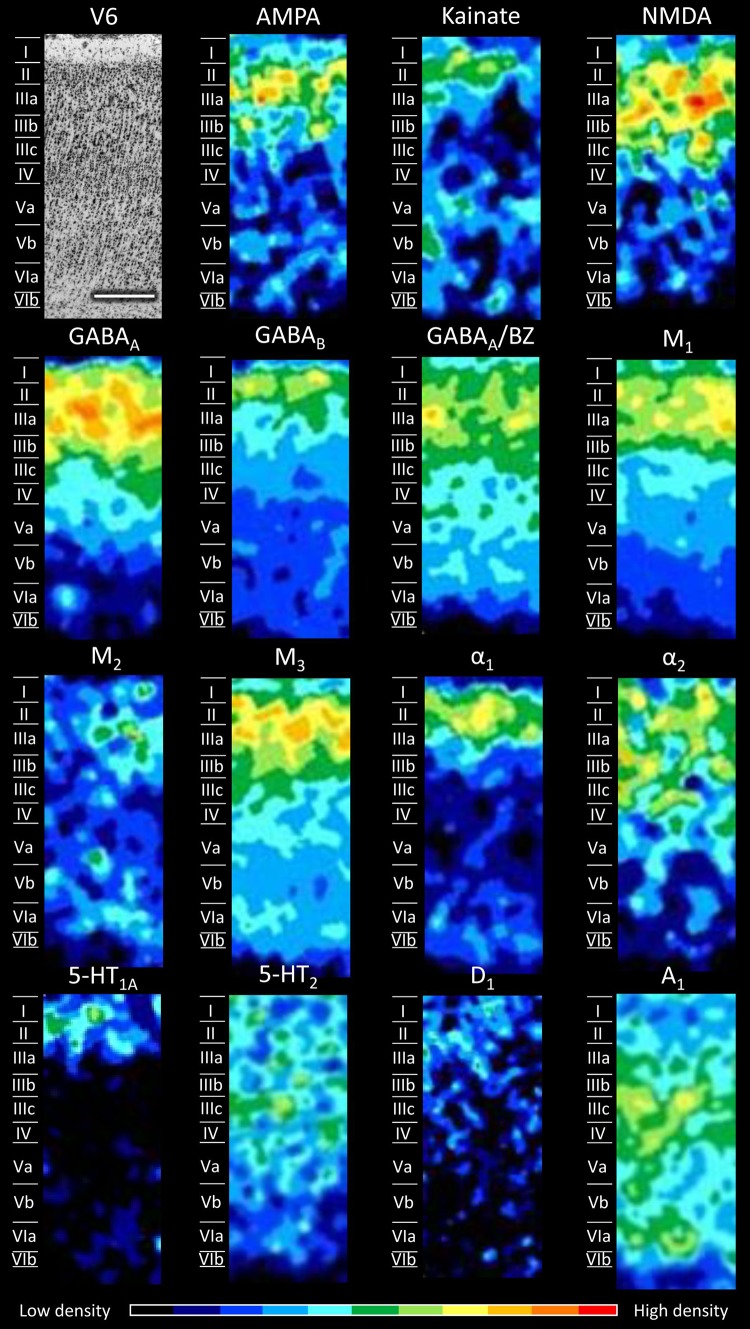
Cyto- and receptor-architecture of area V6. To the left, a Nissl-stained segment is shown. The same segment taken from the corresponding neighbouring autoradiographs is shown for all the 15 receptors analysed. Colour scale codes for receptor densities. Scale bar = 500 µm

**Fig. 6:**
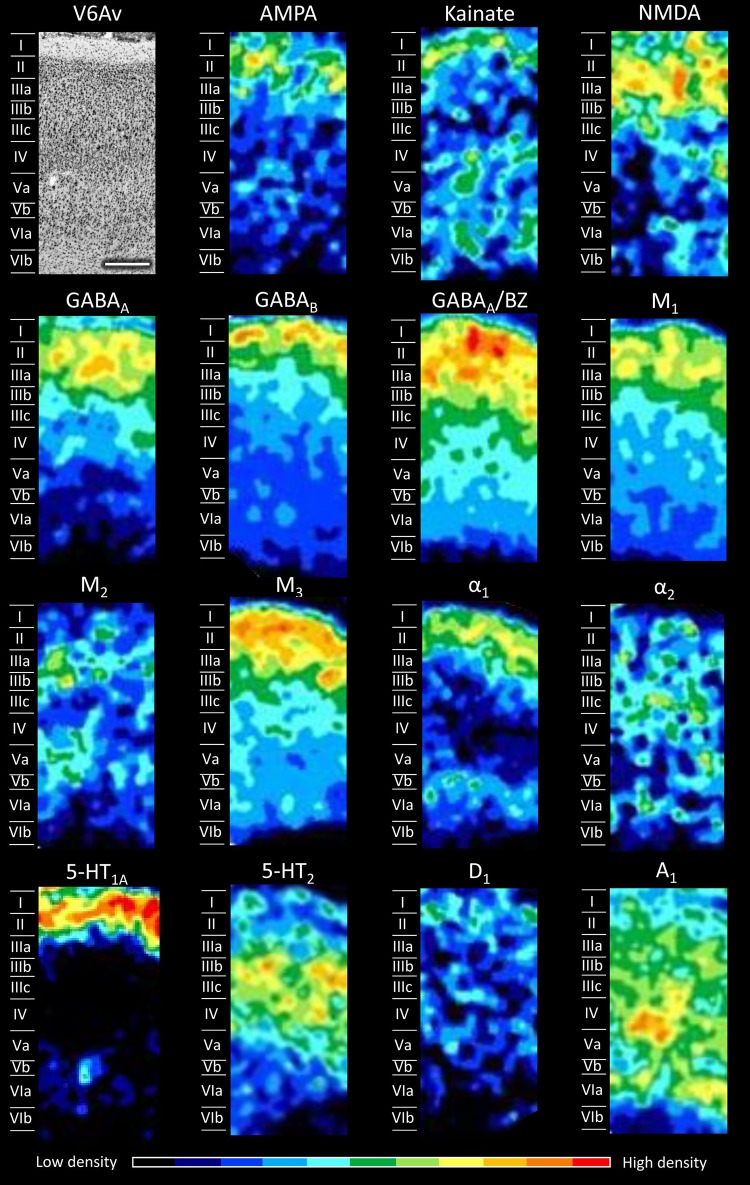
Cyto- and receptor-architecture of area V6Av. Other details in Fig. 5

**Fig. 7:**
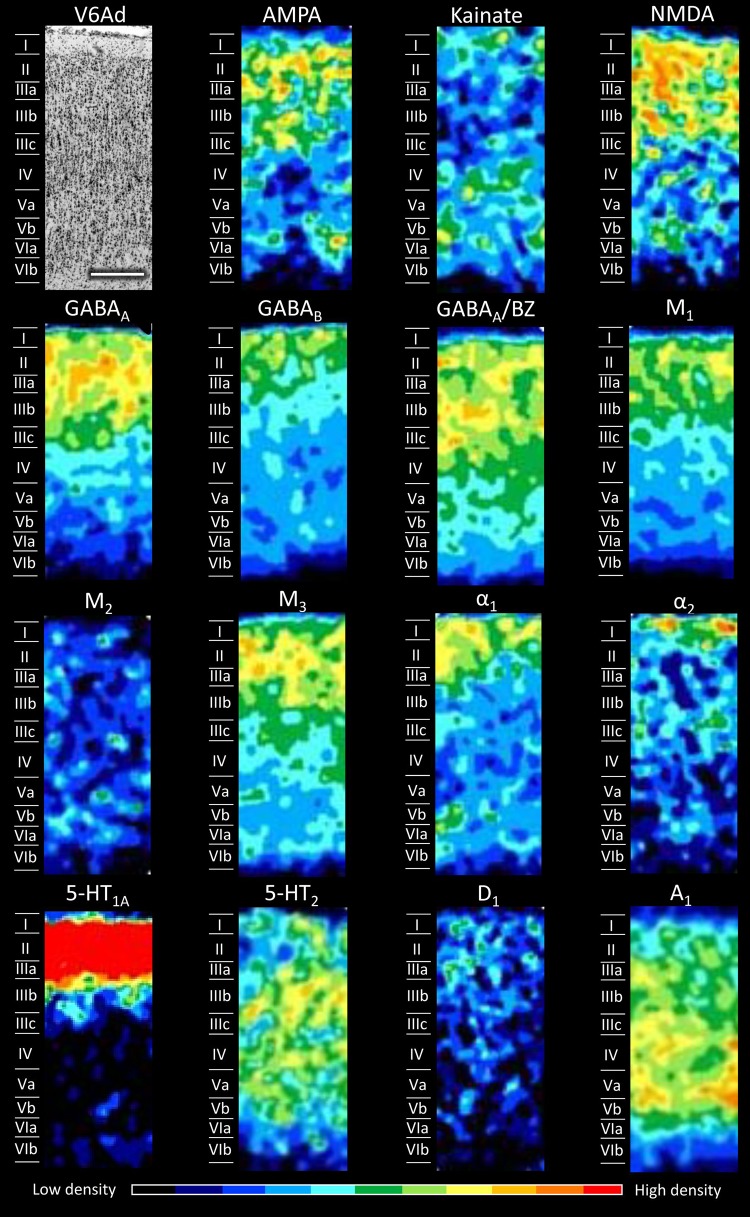
Cyto- and receptor-architecture of area V6Ad. Other details in Fig. 5

**Fig. 8:**
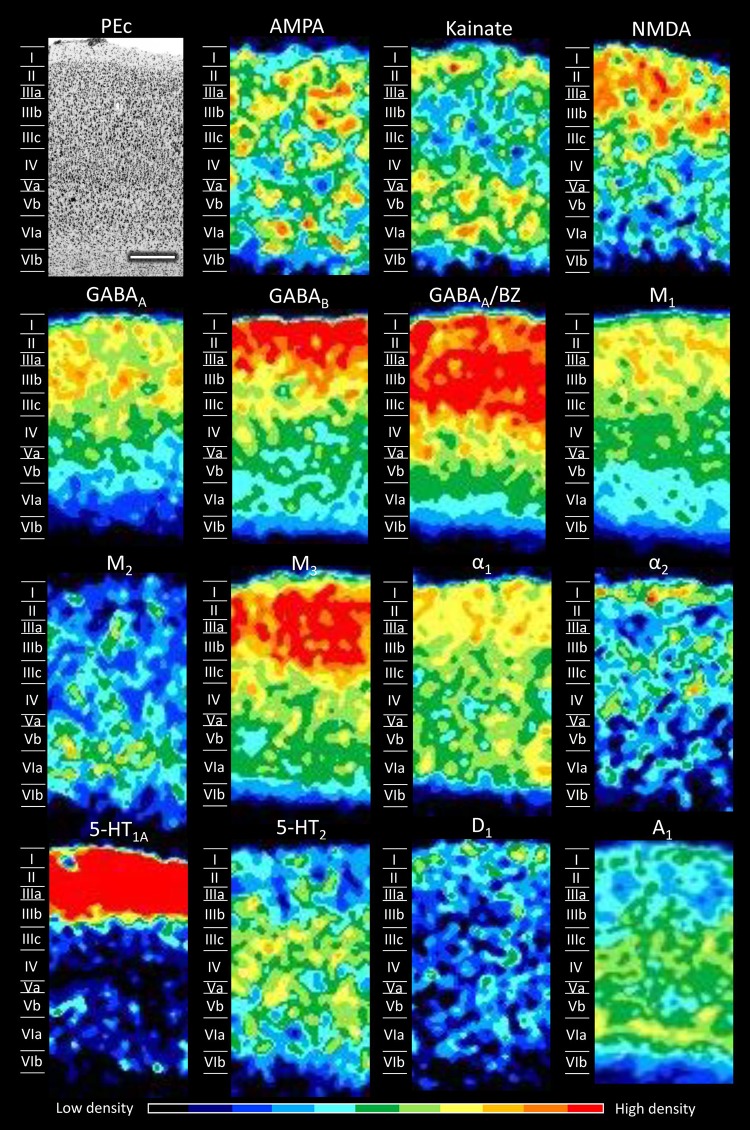
Cyto- and receptor-architecture of area PEc. Other details in Fig. 5

**Fig. 9:**
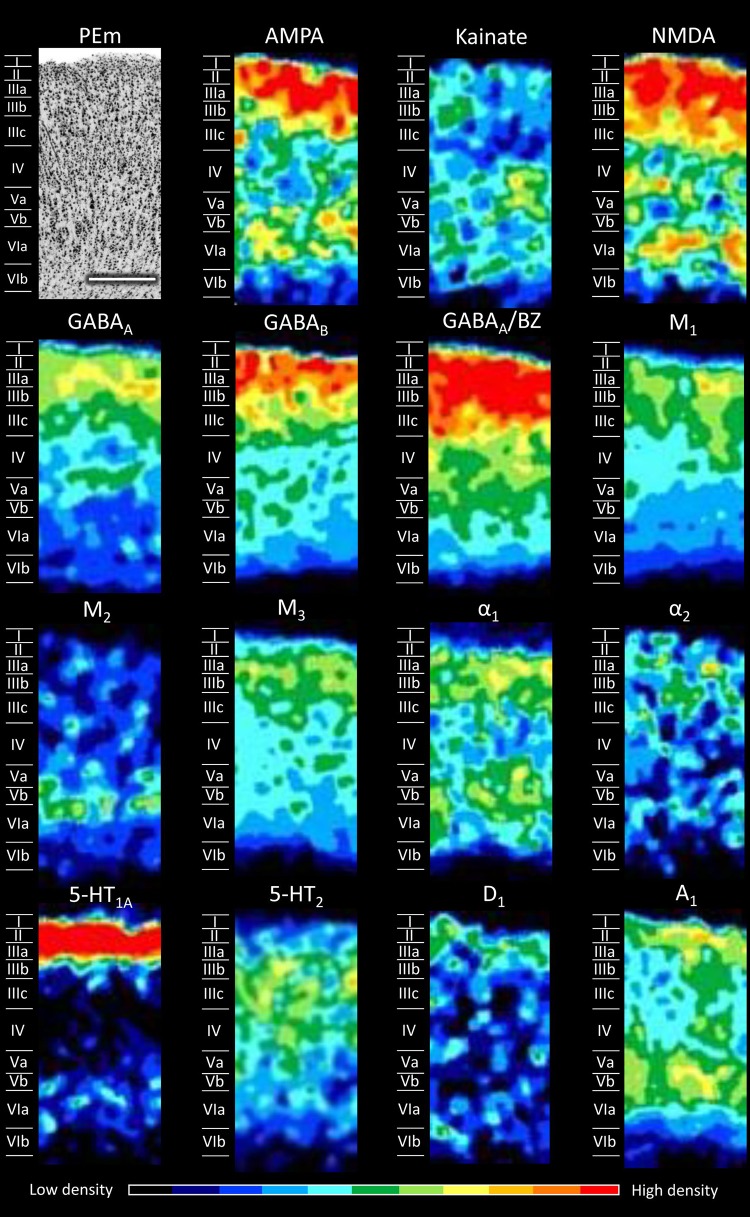
Cyto- and receptor-architecture of area PEm. Other details in Fig. 5

**Fig. 10:**
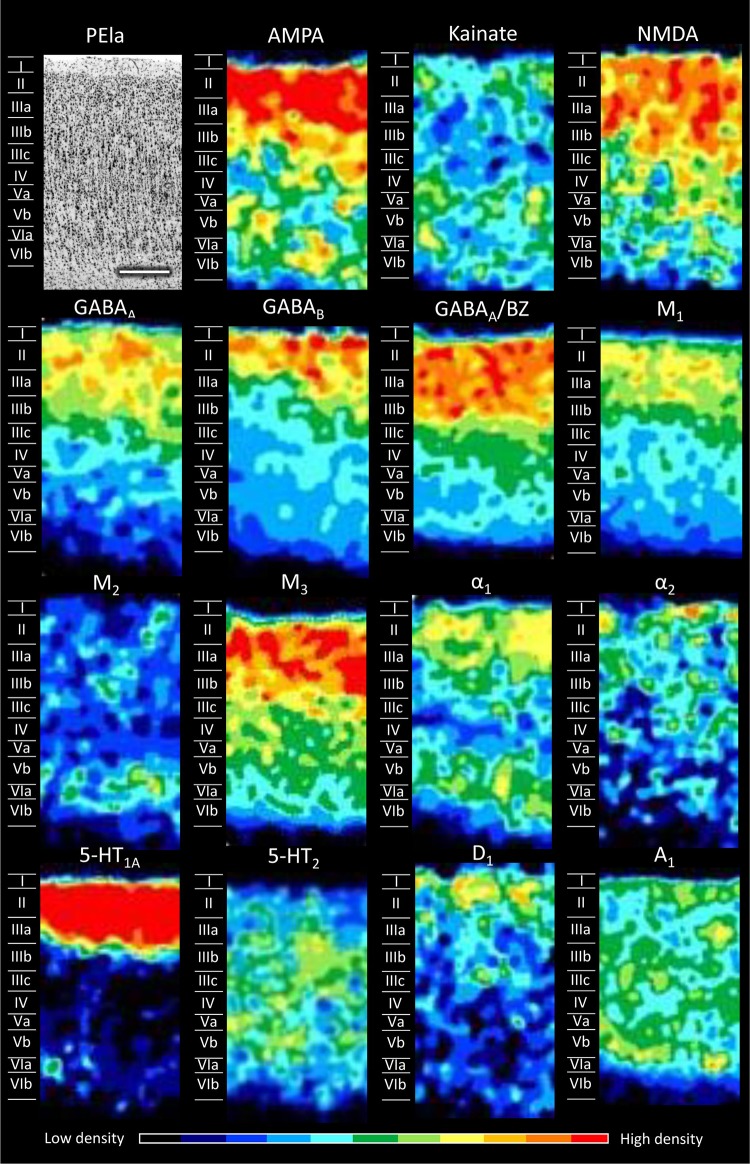
Cyto- and receptor-architecture of area PEla. Other details in Fig. 5

**Fig. 11:**
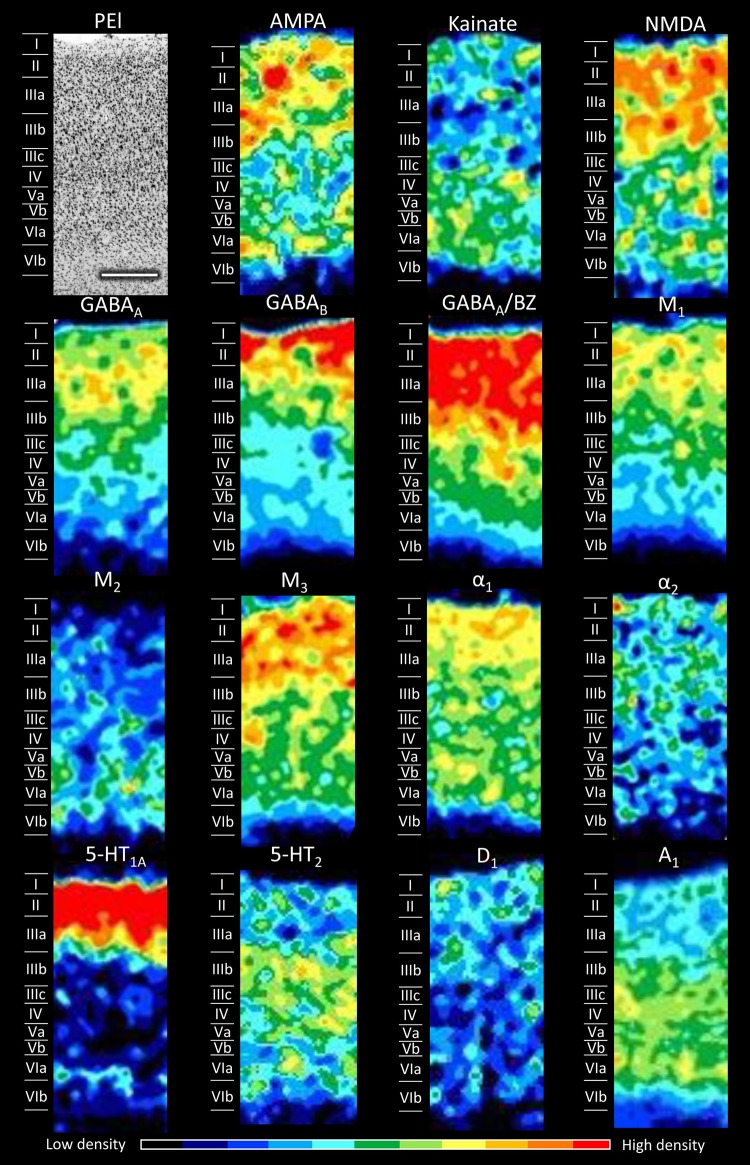
Cyto- and receptor-architecture of area PEl. Other details in Fig. 5

**Fig. 12:**
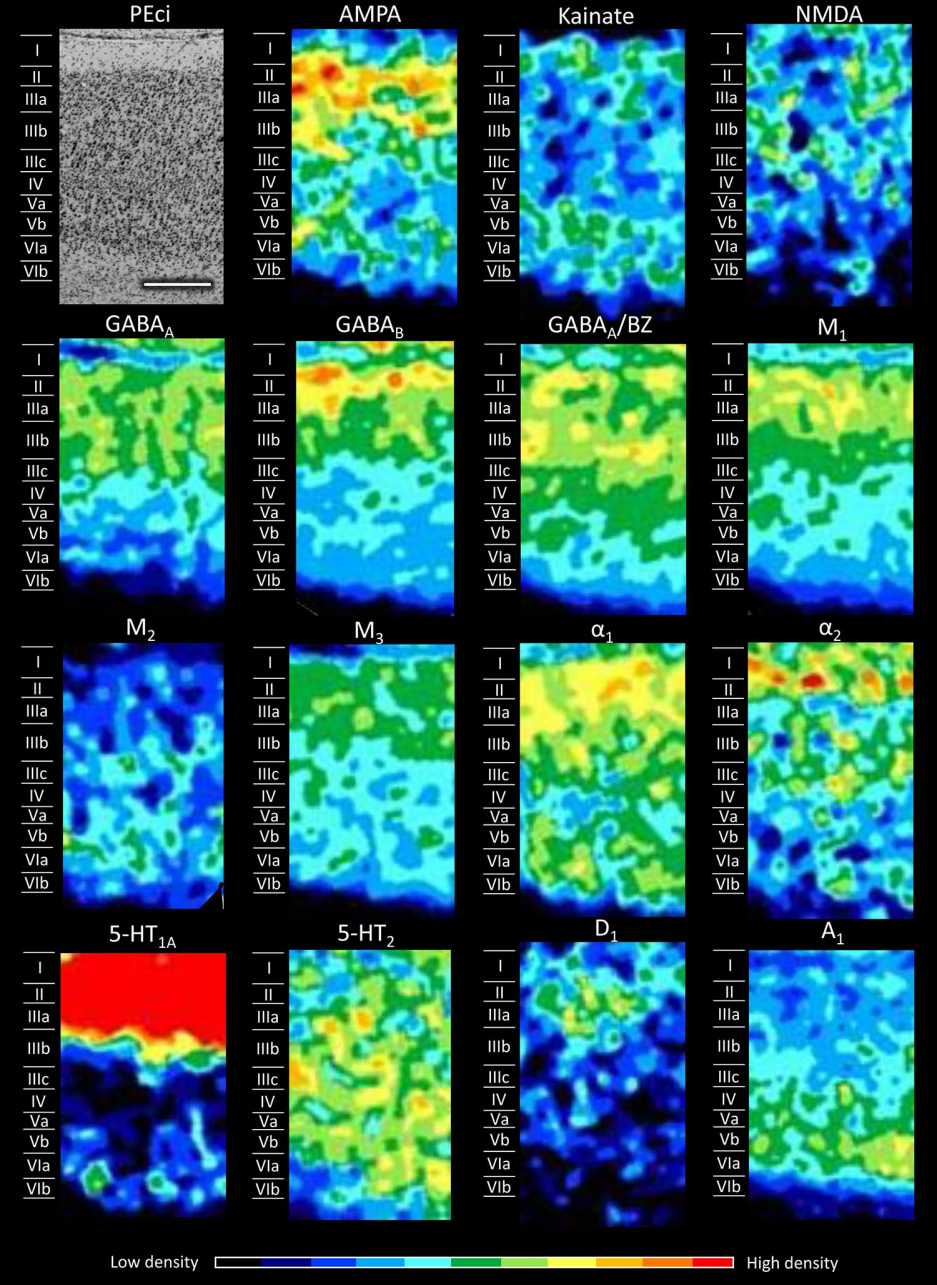
Cyto- and receptor-architecture of area PEci. Other details in Fig. 5

**Fig. 13:**
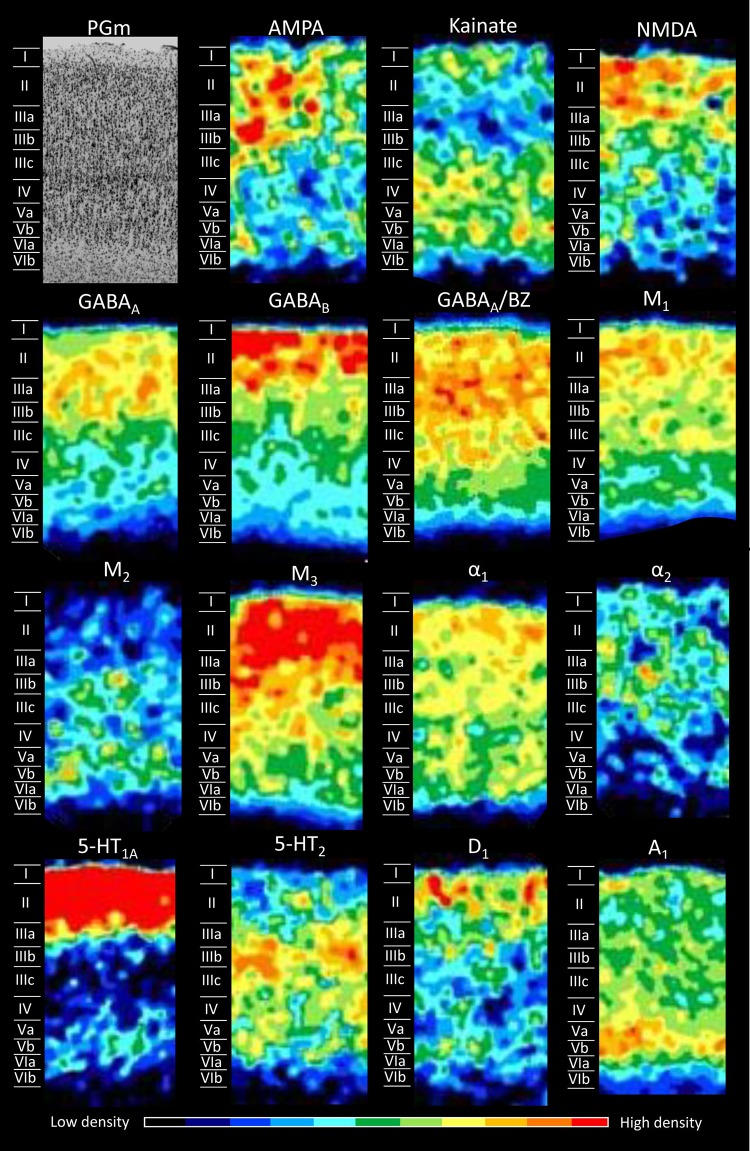
Cyto- and receptor-architecture of area PGm. Other details in Fig. 5

Figures 5, 6, 7, 8, 9, 10, 11, 12, and 13 show the laminar distribution of the 15 receptors analysed in each cortical area of the SPL. At first sight, it is clear that the highest expression level of most receptor types and subtypes is located in the supragranular layers of all areas, although the absolute values reached by each receptor in a specific layer can vary between areas.

#### Areas located at the level of parieto-occipital sulcus (pos)

Three areas are located in the proximity of the parieto-occipital sulcus (pos): areas V6, V6Av, and V6Ad (Fig. 1).

In area V6 (Fig. 5) all receptors reach their maximum expression levels in layers II and/or III. In the glutamatergic family, AMPA and NMDA receptors present the highest densities in layers II and III, whilst receptors for kainate present a local maximum restricted to layer II. GABAergic GABA_A_ receptors and GABA_A_/BZ binding sites show a similar laminar distribution, with highest densities in all the supragranular layers, whereas GABA_B_ receptors are more selective for layer II. Muscarinic M_1_, M_2_, and M_3_ receptors present comparable distribution patterns, since they all reach their maximum expression levels in layers II and III, although the M_2_ density is lower than that of M_1_ and M_3_ receptors. The α-adrenergic receptor of type 1 presents a more restricted distribution compared to the α_2_ receptor, since the former is confined to layers II and IIIa, while the latter is present at considerably higher densities in all supragranular layers than in the infragranular ones. The serotoninergic receptors present a differential distribution pattern, where high 5-HT_1A_ receptor densities are confined to layers I–II, while highest 5-HT_2_ receptor densities are reached layers I–Va, with the highest density in layer III (in particular sublayer IIIc). The D_1_ receptor is present at a very low density throughout the cortex, but there is a higher density in the supra- than in the infragranular layers. The purinergic receptor for adenosine of type 1 (A_1_) shows a different distribution with respect to all the other receptors analysed, since high concentrations were present in layers III–VI, with the highest concentrations in layers IIIc and IV.

**Fig. 14:**
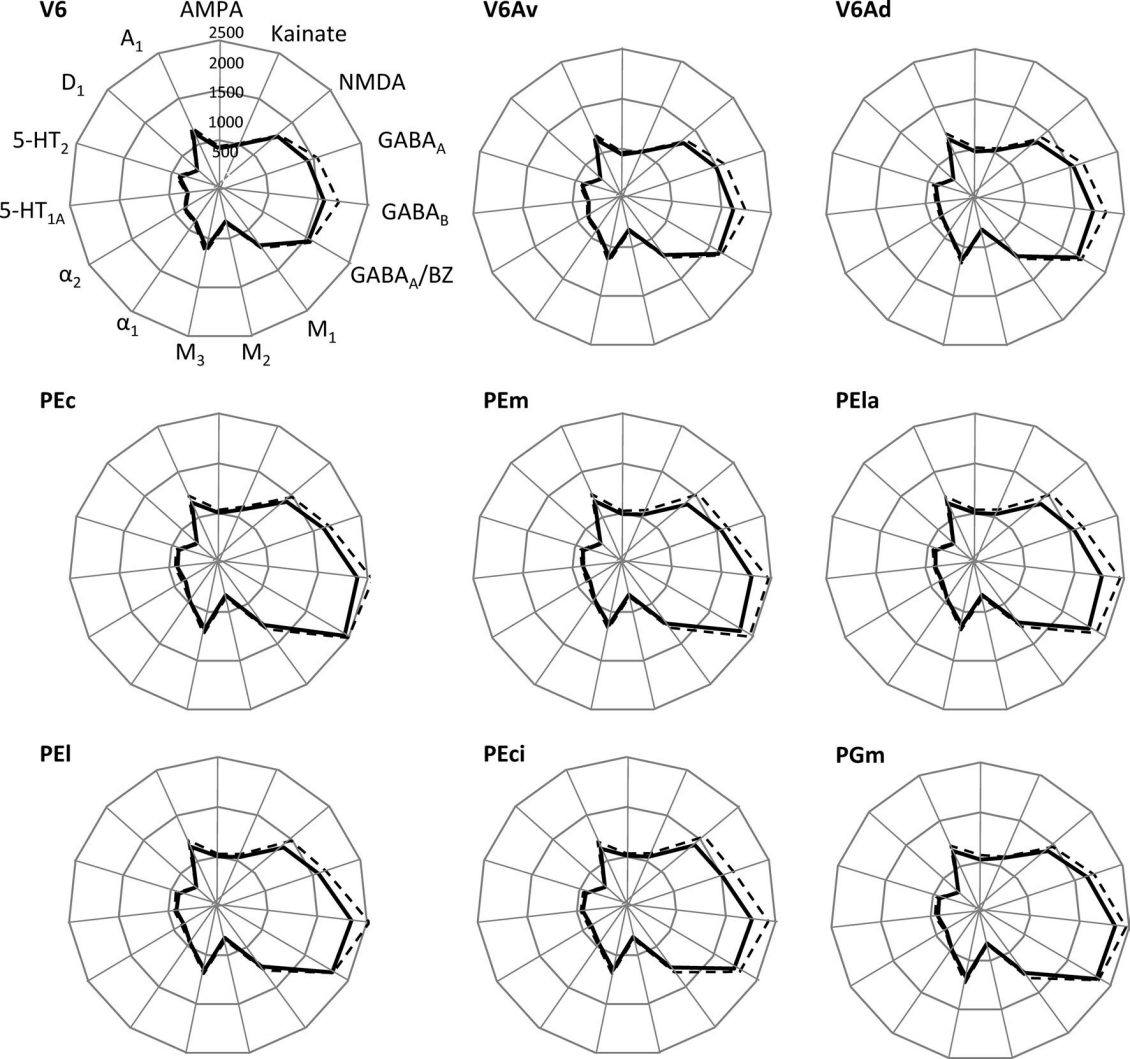
Receptor fingerprints of SPL areas. Continuous lines connect the mean densities and dashed lines the s.d. values. The data are expressed in fmol/mg protein

Area V6Av (Fig. 6) shows a laminar receptor density pattern similar to area V6, although several important differences are evident. At first, the absolute density of most receptors is higher than in area V6 (Fig. 14), and reached the level of significance for the AMPA, kainate, α_1_, and 5-HT_1A_ receptors. Furthermore, highest A_1_ receptor densities are mainly located in the infragranular layers, and the α_2_ receptor presents a more homogeneous distribution throughout all cortical layers.

Area V6Ad is located dorsally to V6Av and close to the exposed surface of the SPL (Fig. 1). In area V6Ad (Fig. 7) the absolute receptor density continues to increase compared to V6Av and V6 (Fig. 14). The densities of AMPA, α_1_, and 5-HT_1A_ receptors, as well as of GABA_A_/BZ binding sites are significantly higher in V6Ad than in V6Av. Differences also exist at the laminar level, the most prominent of which are that in V6Ad the 5-HT_1A_ receptor density is very high at layers II–IIIa, and the highest kainate and A_1_ densities are mainly located in the infragranular layers. Furthermore, M_2_ receptor density is very low and homogeneously distributed throughout all layers of V6Ad, whereas the α_2_ receptor presents a conspicuous maximum in layer I.

#### Areas located on the exposed surface of the SPL

The exposed surface of the superior parietal lobule is composed by two cytoarchitectonic areas, namely PEc and PE (Fig. 1). These two areas differ considerably in their laminar receptor distribution patterns. Furthermore, the receptor-architecture of PEc more closely resembles that of area PGm than it does that of PE. Indeed, PEc and PGm do not differ significantly from each other in the mean (averaged over all layers) densities of any of the examined receptor types, whereas PEc contains significantly higher kainate and α_1_ receptor densities than do the subdivisions of PE.

Area PEc contains significantly higher AMPA, α_1_, and 5-HT_1A_ receptor densities than does V6Ad. The laminar distribution patterns in area PEc (Fig. 8) also differs clearly from those of areas located at the level of the pos, mainly due to higher densities, particularly of AMPA, kainate, and M_2_ receptors, in the infragranular layers of PEc than in those of V6Ad (Fig. 14). The M_2_ receptor presents a local maximum in layer V, and kainate receptors present higher densities in the infra- than in the supragranular layers of PEc. Different from all the other SPL areas, AMPA receptors are homogeneously distributed throughout PEc.

Differences in the laminar distribution patterns and in the mean receptor densities (averaged over all cortical layers; see below) of some of the examined receptors confirmed the subdivision of area PE into areas PEm, PEla and PEl. These three subdivisions of area PE (Figs. 9, 10, 11) present, of course, similarities between them, but important differences are also evident. At the mean receptor level, PEla contains significantly lower AMPA and 5-HT_1A_ densities than PEm and PEl, respectively. Most receptors are present in higher concentrations in the supragranular than in the infragranular layers of all three subdivisions of PE, though exceptions are given by kainate, M_2_, α_1_, and A_1_ receptors. AMPA receptors show a bilaminar distribution pattern in all three subdivisions of PE; however, while in PEm and PEla the densities in the supragranular layers are clearly higher than those in the infragranular layers, in PEl the supragranular layers present only slightly higher densities than the infragranular ones. The NMDA receptor reveals the border between the medial and lateral subdivisions of PE: whereas PEla and PEl present higher densities in the supragranular than in the infragranular layers, PEm shows a second local maximum in layer VI (Fig. 9). The α_1_ receptor enables the delineation of PEla from PEl and PEm, since it does not contain the local minimum over layers IIIc–IV that is clearly visible in the two latter areas. Finally, the A_1_ receptor has a different laminar pattern in each of the subdivisions: in area PEl highest densities extend between layers IIIb and VI, area PEla shows a relatively homogeneous distribution, and in area PEm two local maxima are visible, one involving layers I and II, the other layers V and VIa.

#### Areas located on the mesial surface

On the mesial surface of the hemisphere, two areas were found: area PGm, located in the precuneate cortex, and area PEci, located within the caudal tip of the cingulate sulcus (Fig. 1).

Area PEci (Fig. 12) more closely resembles the subdivisions of area PE, and in particular PEla, than does PGm. PEci and PEm differ significantly in their AMPA and 5-HT_1A_ receptor densities, which are higher in the former area. PEci also contains significantly higher 5-HT_1A_ receptor densities than do PEla or PEl. The main difference between areas PEci and PEla is in their absolute receptor densities (particularly concerning the NMDA, M_3_ and 5-HT_1A_ receptors; Fig. 14), although there are also differences in the laminar distribution pattern of the A_1_ receptor, which in area PEci is present in high concentrations only in the infragranular layers, whilst it is homogeneously distributed throughout area PEla.

As mentioned above, PGm (Fig. 13) is more similar to PEc than to the subdivisions of PE. Mean receptor densities of PGm do not differ significantly from those measured in PEc. Furthermore, densities of NMDA, GABA_A_, α_1_, and 5-HT_2_ receptors are comparable in PEm, PEla and PEl, but conspicuously lower than those of PEc or PGm. Additionally, PGm shows a laminar receptor pattern which is very similar to that of PEc. All the receptors are mainly expressed in supragranular layers, with the exception of kainate, M_2_, 5-HT_2_ and A_1_ receptors, showing a reverted pattern. Major differences between PGm and PEc are visible only for the laminar distribution patterns of AMPA, α_2_ and D_1_ receptors. The density of AMPA receptors is widespread in PEc but mainly concentrated in the supragranular layers in PGm. Another difference regards α_2_ receptor, confined in layer III in PGm, whilst in PEc (Fig. 8) it is organized in two bands, one denser at the level of layer I and another one in correspondence of layer III, with a low-density zone in layer II. An additional difference is visible comparing D_1_ receptor, which presents a low density even if shifted towards layer I in PEc, but reaching high amounts in PGm, and more with the involvement of layers II and IIIa.

### Receptor fingerprints

Figure 14 summarizes the data of the mean density (averaged over all cortical layers) for all the receptors analysed for each area. All areas share the common characteristic that GABAergic and NMDA receptors are the most highly expressed types in all areas. Furthermore, M_1_, M_3_ and A_1_ receptors are present at higher densities than the remaining receptor types, and the D_1_ receptor is present at the lowest concentration. Area V6 has the smallest receptor fingerprint, thus highlighting the fact that it contains the lowest mean densities of receptors measured within areas of the SPL, and areas PEc and PGm have the largest receptor fingerprints. Figure 14 shows how the increase in absolute densities observed when moving from V6 through V6Av to V6Ad and PEc is reflected in an increase in the size of their respective fingerprints.

The MANOVA revealed that, under simultaneous consideration of all receptor types, SPL areas differ significantly from each other, and the subsequent ANOVAs identified the changes in the densities of AMPA, kainate, α_1_ and 5-HT_1A_ receptors, as well as of GABA_A_/BZ binding sites as the main differences driving this significance.

**Fig. 15:**
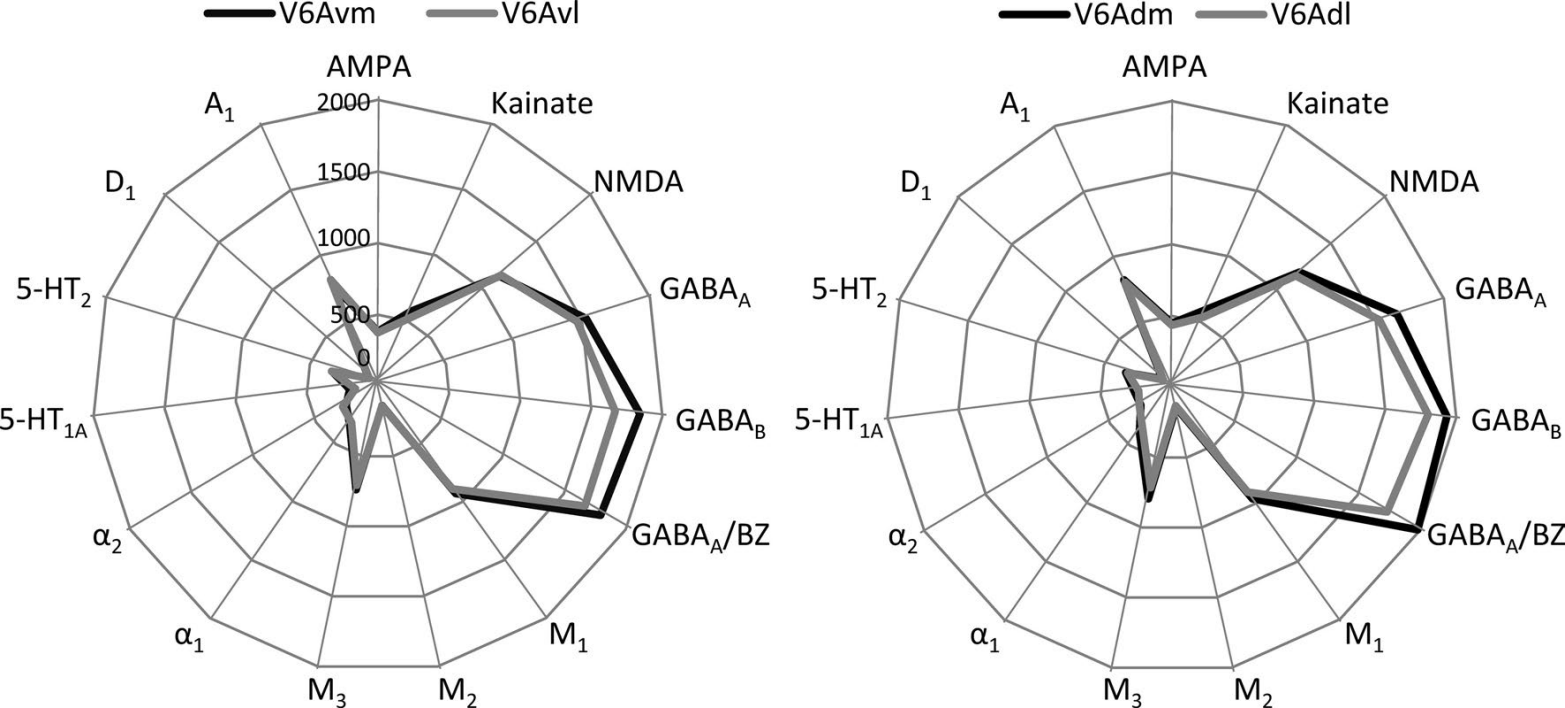
Receptor fingerprints of the medial and lateral subdivisions of areas V6Av and V6Ad. Continuous lines connect the mean densities and dashed lines the s.d. values. The data are expressed in fmol/mg protein

The analysis of mean receptor densities also allowed us to identify medial and lateral subdivisions within areas V6Av and V6Ad. Indeed, receptor densities are higher in the medial than in the lateral parts of the two areas (Figs. 2 and 15). In the case of areas V6Avl and V6Avm, this difference reached significance for the kainate receptors, whereas V6Adl and V6Adm differed significantly in their 5-HT_1A_ densities. However, we observed that these differences in mean densities were not accompanied by differences in cytoarchitecture or in the laminar distribution patterns of the examined receptors.

#### Multivariate analyses

**Fig. 16:**
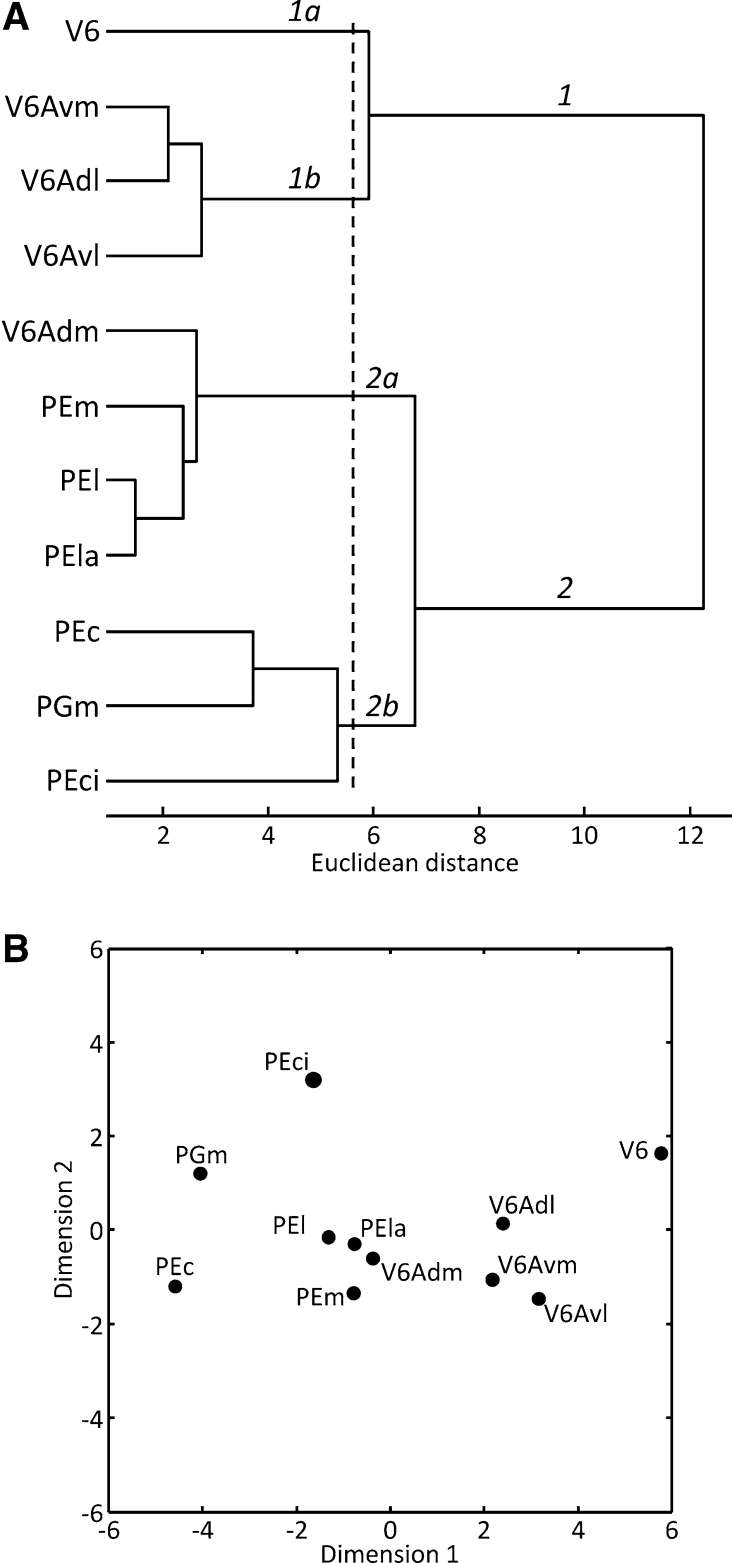
Hierarchical cluster (**a**) and multi-dimensional scaling (**b**) analyses of the mean receptor densities of areas of the SPL

Multivariate analyses were carried out to assess the degree of (dis)similarity of the receptor fingerprints of areas of the SPL. The *k*-means analysis revealed that areas of the SPL could be divided into four clusters based on their receptor fingerprints. The hierarchical cluster analysis revealed a first clear separation of areas V6, V6Avl, V6Avm and V6Adl (branch 1; Fig. 16a) from area V6Adm and the areas on the lateral and medial aspects of the SPL (branch 2; Fig. 16a). Furthermore, area V6 builds a cluster on its own (branch 1a; Fig. 16a), whereas areas V6Avl, V6Avm and V6Adl are located within one cluster (branch 1b; Fig. 16a). This clear segregation of area V6 from V6Ad and V6Av is also confirmed by the multidimensional scaling analysis (Fig. 16b). Interestingly, V6Adm is found in the same cluster as areas PEm, PEl and PEla (branch 2a; Fig. 16a). In the multidimensional scaling analysis, V6Adm takes an intermediate position between the subdivisions of area PE and areas V6Avl, V6Avm and V6Adl (Fig. 16b). Finally, areas PEc, PEci and PGm are found in a common cluster of the dendrogram (branch 2b; Fig. 16a), but are clearly segregated by the second dimension of the multidimensional scaling analysis (Fig. 16b).

## Discussion

The present study constitutes a multimodal analysis of areas located in the SPL, encompassing both their cytoarchitecture and multi-receptor expression patterns. This approach not only enabled the definition of hitherto undescribed borders within the SPL, namely the subdivisions of area PE, but also provided new insights into the functional organization of SPL areas. Based on the present findings, and in view of recent advances in our understanding of the structural and functional organization of the primate superior parietal lobule, we here propose a novel pattern of homologies between areas of the human and macaque SPL (see below).

The first insight resulting from the receptor density analysis is that receptors for GABA and glutamate are the predominant receptor types in all the areas of the SPL. This finding is in line with observations in other macaque brain regions such as the primary sensory (Zilles and Palomero-Gallagher 2017) and cingulate (Bozkurt et al. 2005; Palomero-Gallagher et al. 2013) cortices, as well as with analyses of homologue regions in the human brain (Eickhoff et al. 2007; Palomero-Gallagher et al. 2009, 2013; Scheperjans et al. 2005a, b; Zilles and Palomero-Gallagher 2017). Furthermore, it highlights the role that both neurotransmitters play, together with modulatory neurotransmitters, in maintaining the balance between excitation and inhibition essential for a correct functioning of the brain (Markram et al. 2004; Rao et al. 2000; Wehr and Zador 2003; Wu and Sun 2015; Xu et al. 2011).

### Parcellation schemes of the SPL

The parietal cortex has been object of several cytoarchitectonic studies, some of which have provided maps of the entire SPL (Brodmann 1909; Lewis and van Essen 2000; Morecraft et al. 2004; Pandya and Seltzer 1982), whereas others have focussed on specific parts such as the cortex located within the anterior wall of the parieto-occipital sulcus (Colby et al. 1988; Luppino et al. 2005) or the precuneus (Passarelli et al. 2018). Our multivariate analysis confirms the parcellation scheme proposed by Luppino et al. (2005) for the anterior bank of the pos, and the existence of an area PGm in the precuneus (Pandya and Seltzer 1982; Passarelli et al. 2018). We largely confirmed the delineations of Morecraft and colleagues (2004) on the convexity of the SPL and within the cingulate sulcus, but found subdivisions within their area PE. Finally, analysis of the GLI profiles enabled us to revise the identification of cortical layers and sublayers of the SPL areas, and the identification of three sublayers for layer III, and two sublayers for layer V.

Both the quantitative analysis of cytoarchitectonic features (GLI analysis) and the qualitative and quantitative assessment of the regional and laminar distribution patterns of multiple receptor types corroborated the subdivision of area V6A into dorsal and ventral components in the anterior bank of the pos, as well as the location and extent of area PGm in the precuneus. Furthermore, we found that the medial part of areas V6Av and V6Ad presented a higher receptor density pattern compared to the lateral part, in particular for GABA receptors. This may be explained by the fact that the medial parts of both areas are involved in further, or different, processes with respect to the medial ones. Specifically, since the precuneate cortex anterior to V6A codifies for complex actions related to spatial navigation, scene perception, and spatial working memory (Baumann and Mattingley 2010; Hutchison et al. 2015; Kravitz et al. 2011; Sato et al. 2006, 2010), it can be suggested that the higher receptor density found in the medial part of areas V6Av and V6Ad could be necessary to encode complex aspect of the visuo-motor integration. Also, it has been recently demonstrated that V6A is active during covertly shifts of spatial attention (Caspari et al. 2015; Galletti et al. 2010). Very recent fMRI experiments show that during attentional shifts two separate foci of activation appear in the medial and lateral parts of macaque V6A (see Fig. 3b of Caspari et al. 2018), suggesting possible different functional role for these two brain regions.

We were also able to confirm the existence of areas PEci, previously described by Morecraft and colleagues (2004), and PE (Pandya and Seltzer 1982). However, while originally Pandya and Seltzer (1982) defined area PE as a cytoarchitectonic homogeneous entity, we have found here three subdivisions within PE. It is worthwhile to notice that recent studies also focussed on connectional characteristics have pointed at possible subdivisions within area PE (Bakola et al. 2013; Gamberini et al. 2017; Impieri et al. 2018), and recent functional studies have also shown that area PE is divided into different somatotopic sectors (see, for example, Seelke et al. 2012). Figure 4b shows the three subdivisions of area PE we have found here: area PEl is visible on the exposed surface of the SPL as a relatively thin strip on the rim of the intraparietal sulcus, and continues into the outer portion of the medial bank of the intraparietal sulcus area PEla forms a long strip in front of area PEl, and PEm is a cortical region located dorsomedially. PEla and PEl seem to overlap with the somatotopic map shown by Seelke and coworkers in area 5 [see Fig. 9 of Seelke et al. (2012)], while the region we defined as PEm still needs to be functionally characterized. Connectional studies reported that the lateral part of PE receives preferential input from anterior sectors of the medial bank of intraparietal sulcus and from the ventral premotor cortex, whereas medial part of PE forms denser connections with area PEc and motor fields. In conclusion, both functional and connectional studies support the view of subdivisions within area PE, but further experiments are needed to determine the functional role(s) of these subdivisions.

### Receptor-architectonic and functional organization of SPL areas

Comparing the receptor fingerprints of the SPL areas, a gradual increase in their size, which reflects an increase in receptor densities, is appreciable when moving from the fundus of the pos to the exposed surface of the SPL. This increase in receptor density can be associated with differences in the functional characteristics of the areas of the SPL. Visual and somatosensory cells are located in the SPL cortex with a distinctive differentiation depending on the cortical area examined (Gamberini et al. 2011, 2015, 2018; Mountcastle et al. 1975; Seelke et al. 2012). In area V6 all cells are responsive to visual stimuli, while in V6Av and V6Ad only a part of them are visually responsive, while others are responsive to somatosensory stimuli are present (20 and 40% of the total amount in V6Av and V6Ad, respectively; Gamberini et al. 2011). In area PEc, cells responsive to somatosensory stimuli slightly prevail over those responsive to visual stimuli, reaching 60% of the total amount (Gamberini et al. 2018). Area PE, instead, represents the somatic counterpart of area V6, in the sense that almost the totality of PE neurons (99%) are activated by somatosensory or somatomotor stimulations (Mountcastle et al. 1975). No detailed findings on cellular properties are available for areas PEci or PGm. However, from the available studies (Murray and Coulter 1981 for area PEci; Leichnetz 2001; Olson et al. 1996; Passarelli et al. 2018; Thier and Andersen 1998 for area PGm), it is possible to assume that area PEci contains mainly somatosensory neurons, while neurons responding to both visual and somatosensory stimuli can be found in area PGm. Viewed as a whole, two functional streams seem to coexist in the SPL: a visual one, whose intensity decreases moving from the fundus of the pos to the exposed surface of the SPL, and a somatosensory one, whose intensity decreases in the opposite direction. While V6 and PE are unimodal areas, PEc, PGm, and V6Ad, that are central nodes of these two streams, are bimodal visual-somatic areas. PEc and PGm are also the areas with the highest receptor densities of all SPL areas, and V6Ad has the largest fingerprint of all areas located within the pos. We believe that the high receptor density represents an important physiological base for the control of limb movement in reaching and grasping activity. In fact, these activities require a fine and precise integration of visual and somatic stimuli, particularly proprioception and tactile information coming from the limbs, as it is the case in both areas PEc and V6Ad (Gamberini et al. 2018). High receptor densities, particularly of GABAergic and NMDA receptors as shown in the present results, could provide the specific balance of excitatory and inhibitory neurotransmissions needed to control the limb movements during visually guided actions. The bimodal SPL areas PEc and V6Ad (and maybe PGm) located at the interface between somatic and visual system could play a crucial role in the visual guidance of limb movements (Gamberini et al. 2018).

### Comparison with the human SPL

The human SPL encompasses Brodmann’s areas 5 and 7 (Brodmann 1909), which are equivalent to area PA_2_ and areas PE_m_, PE_p_ and PE_γ_ of von Economo and Koskinas (1925), respectively. More recent studies applying quantitative cyto- and receptor autoradiographic techniques revealed that Brodmann’s area 5 is composed of three subdivisions, i.e. 5L, 5M, and 5Ci, whereas his area 7 was divided into 4 parts, i.e. 7A, 7P, 7PC, 7M (Scheperjans et al. 2005a, b, 2008a, b).

In the light of present results, we suggest that macaque area PEci corresponds to human area 5Ci, macaque areas PEla and PEl, together, to human area 5L (see above in “Discussion”), and PEm is the homologous of human 5M. As far as Brodmann’s area 7 is concerned, it occupies most of the exposed surface of SPL in humans, and has been divided into four cyto- and receptor-architectonically distinct areas (areas 7A, 7P, 7PC, 7M; Scheperjans et al. 2008a). In macaque, on the contrary, area 7 is reported in the mesial surface of the hemisphere (area PGm) and in the IPL (PG, PFG, PF), with a very thin and often disregarded cortical strip in the caudal most part of the exposed surface of SPL. While human area 7M is likely the homologous of macaque area PGm, for a number of reasons it seems to us unlikely that areas 7A, 7P, 7PC are the homologs to areas PG, PFG, PF. Since we have suggested that area PEc, in the caudal aspect of macaque SPL, is part of area 7 (see above in “Discussion”; Galletti and Gamberini 2018; Gamberini et al. 2017), we believe that the caudal part of human area 7 could be the homologous of macaque area PEc of von Economo and Koskinas (1925). Further studies are needed to verify this hypothesis.

For the areas of the pos, studies in humans that used autoradiographic techniques have not yet reached the same degree of accuracy as in the macaque. So far, several ventral and dorsal parts have been identified within Brodmann’s area 19 (e.g. hOc3d, hOc3v, hOc4d, hOc4v; Kujovic et al. 2013; Rottschy et al. 2007) with different receptor-architectonic patterns (Scheperjans et al. 2005b). At the same time, functional studies in human allowed to recognize the homologues of macaque areas V6, V6Av, and V6Ad (Pitzalis et al. 2006, 2013; Tosoni et al. 2015; for a review also see Pitzalis et al. 2015). However, to date, we have no hints to suggest a parallel in the human between the cytoarchitectonically or receptor-based defined areas within Brodmann’s area 19 and the functionally defined areas V6, V6Av, and V6Ad. Again, further studies are needed to clarify this point.

## Concluding remarks

The present multivariate analysis of receptor fingerprints confirms the associative role of SPL areas in the encoding of visual and somatosensory stimuli necessary to execute reaching and grasping movements (Fattori et al. 2017; Galletti and Fattori 2018; Galletti et al. 2003; Gamberini et al. 2011; Mountcastle et al. 1975; Seelke et al. 2012). Based on differences in cytoarchitecture and laminar receptor distribution patterns, we were able to identify and characterize novel subdivisions of area PE and provide new insights into the functional organization of the macaque SPL. The data reported here support a good homology between macaque and human SPL. Hopefully, future analyses will elucidate whether the ensuing novel parcellation scheme of the SPL has reliable functional counterparts, as suggested here.
